# Analysis of Security Vulnerabilities in S-100-Based Maritime Navigation Software

**DOI:** 10.3390/s26041246

**Published:** 2026-02-14

**Authors:** Hoyeon Cho, Changui Lee, Seojeong Lee

**Affiliations:** 1 Division of Maritime Information Technology, National Korea Maritime and Ocean University, Busan 49112, Republic of Korea; hoyeoncho@g.kmou.ac.kr; 2 SemanticWave, Busan 47257, Republic of Korea; 3 Division of Marine System Engineering, National Korea Maritime and Ocean University, Busan 49112, Republic of Korea; culee@kmou.ac.kr

**Keywords:** S-100 standard, maritime cybersecurity, remote code execution, static analysis, portrayal engine, Lua scripting

## Abstract

The S-100 standard for Electronic Chart Display and Information Systems (ECDIS) uses Lua scripts to render electronic charts, yet lacks security specifications for script execution. This paper evaluates automated Static Application Security Testing (SAST) tools versus expert manual review for S-100-compliant software. Four SAST tools were applied alongside an expert review of OpenS100, a reference implementation for next-generation ECDIS. While automated tools identified numerous defects, they failed to detect 83% (19/23) of expert-identified vulnerabilities, including an unrestricted Lua interpreter flaw with a Common Vulnerability Scoring System (CVSS) score of 9.3. This vulnerability enables Remote Code Execution (RCE) via malicious portrayal catalogues, verified through Proof of Concept (PoC) development. The analysis demonstrates that SAST tools are constrained by limited maritime domain knowledge and challenges in analyzing cross-language semantic risks at the C++–Lua interface. The findings establish that identified vulnerabilities stem from specification gaps in the S-100 standard rather than isolated coding errors. These results indicate that functional safety certifications require supplementation to address design-level security risks. The evidence supports that the International Hydrographic Organization (IHO) incorporate security controls, such as script sandboxing and library restrictions, into the S-100 framework before the 2029 mandatory adoption deadline.

## 1. Introduction

Maritime navigation has evolved from paper nautical charts to Electronic Navigational Charts (ENC), with the International Hydrographic Organization (IHO) introducing the S-100 standard to replace the legacy S-57 format [1]. The S-100 standard separates data from visualization through a portrayal engine that interprets Lua scripts to render Electronic Chart Display and Information Systems (ECDIS). While S-100 includes cryptographic mechanisms such as IEC 63173-2 SECOM (Secure Communication Between Ship and Shore) along with Part 15 digital signatures ensuring chart data originate from authorized hydrographic offices, cryptographic integrity guarantees source authenticity but does not guarantee script safety [2,3]. Malicious portrayal scripts from compromised sources could manipulate chart rendering, leading to navigation hazards or operational disruptions. Modern operating system protections such as Data Execution Prevention (DEP) defend against memory attacks but may offer limited effectiveness against vulnerabilities in how software interprets scripts for standard compliance.

Commercial security analysis tools effectively detect common software defects, such as buffer overflows, but they face limitations when different programming languages interact. The S-100 portrayal engine exemplifies this challenge, as navigation-critical logic flows between C++ chart parsing and Lua script execution. This analysis gap represents a functional safety concern as the maritime industry transitions from S-57 to S-100. Existing maritime cybersecurity research addresses network attacks or equipment firmware vulnerabilities but has not examined security implications of navigation data standards requiring script interpretation.

This paper employs a three-phase methodology to quantify this analysis gap. In Phase 1, the analysis target and build environment were established. In Phase 2, four static analysis tools representing typical security verification practices including Microsoft Visual Studio (MSVC), CppCheck, FlawFinder, and CodeQL were applied to an S-100-compliant ECDIS implementation, identifying numerous software defects. However, none of these automated findings identified portrayal script vulnerabilities capable of compromising navigation displays. This result indicates a gap in conventional tools when applied to maritime software with embedded scripting components. In Phase 3, expert manual review examined the C++–Lua interface logic, identifying design flaws that could enable malicious Portrayal Catalogues (PC) distributed through ENC update channels to execute unauthorized commands by exploiting the trust boundary between cryptographically verified data and unvalidated scripts.

A critical distinction should be noted: one of the identified vulnerabilities originates from structural gaps in the S-100 standard specification rather than implementation-specific defects in the analyzed system. The S-100 standard Parts 9a and 13 mandate Lua 5.1 usage for PC implementation [4,5]. However, they provide no security requirements for script execution environments, such as sandboxing specifications, library restrictions, or capability limitations. This absence of security guidance creates a scenario where implementations following the standard’s functional requirements correctly may nonetheless introduce security vulnerabilities. The analyzed OpenS100 system serves as a reference implementation that influenced multiple derivative systems, making the identified vulnerabilities indicative of broader ecosystem risks rather than isolated implementation failures. S-100-compliant implementations that follow the standard’s scripting requirements without adding implementation-specific security controls may be susceptible to similar architectural risks, though verification across commercial ECDISs remains necessary for comprehensive assessment.

This paper makes three contributions. First, to the best of our knowledge, it provides one of the first empirical security evaluations of S-100 implementations through quantitative analysis comparing automated and manual detection capabilities. Second, it quantifies the semantic gap in automated security analysis, where automated findings showed limited effectiveness in detecting design-level architectural vulnerabilities despite extensive code coverage. Third, it establishes that the identified vulnerabilities are associated with the S-100 portrayal engine design, indicating that functional safety certification for next-generation ECDIS may benefit from including domain-specific verification to address security considerations. Section 2 reviews background and related work. Section 3 describes methodology. Section 4 presents findings and Section 5 shows the case study. Section 6 addresses limitations and further research. Section 7 concludes this study.

## 2. Background and Related Work

### 2.1. S-100 Standard and Portrayal Engine Architecture

The IHO S-100 Universal Hydrographic Data Model provides the technical foundation for next-generation ECDIS. S-100 was developed to overcome the rigid data structure and maintenance limitations of the legacy S-57 standard and is designed to align with the ISO 19100 series of geographic information standards [1]. The standard extends beyond simply defining encoding formats by providing a flexible framework for developing various marine-related data product specifications based on object-oriented modeling using Unified Modeling Language.

From a technical perspective, a key architectural change in S-100 is the separation of data features from portrayal visualization logic. Unlike legacy systems that relied on static symbol tables and hardcoded visualization rules, S-100 defines a portrayal mechanism in Part 9 (Portrayal), Part 9a (Portrayal-Lua), and Part 13 (Scripting). Specifically, S-100 Edition 5.2.0 Part 9a Section 9a-4.1 mandates: “*Lua version 5.1 shall be used for implementing portrayal functions*” [4]. Additionally, Part 13 Section 13-7.1 specifies the Lua version requirements [5]. PCs consist of XML files containing rendering rules and Lua scripts for their execution, which systems dynamically load at runtime to render maritime objects on displays. The IHO GI Registry serves as the authoritative metadata repository for S-100 product specifications, while FCs and PCs, as integral components of international standards, are distributed through official IHO channels and national hydrographic offices, establishing a centralized distribution model with potential supply chain implications.

**Figure 1:**
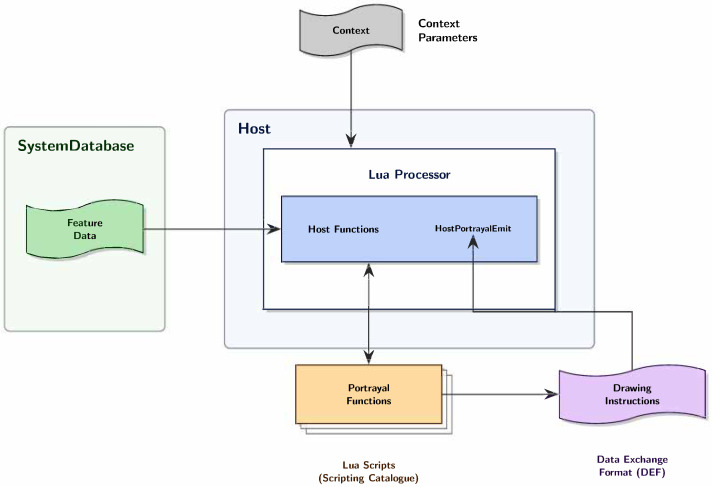
S-100 Part 9a Lua portrayal architecture and data flow (adapted from [4]).

Figure 1 illustrates the S-100 Part 9a Lua portrayal architecture and data flow between system components. The portrayal process operates through a clearly defined pipeline where the System Database stores Feature Data describing maritime objects including navigation aids, depth contours, and hazards. The Host application, implemented in C++, instantiates a Lua Processor that loads and executes Portrayal Functions from the Scripting Catalogue.

The execution model establishes a bidirectional communication channel between the C++ host and Lua scripts. Host Functions provide callback mechanisms allowing Lua scripts to query feature attributes, spatial relationships, and information associations from the Feature Data. Context Parameters such as display scale, safety depth settings, and viewing modes are passed into the Lua environment to control rendering behavior. The Lua scripts process this information and return Drawing Instructions in Drawing Exchange Format (DEF) via the *HostPortrayalEmit* callback function, which the host then renders on the display.

This architecture creates a critical trust boundary at the C++–Lua interface. While Feature Data undergoes cryptographic verification through Part 15 digital signatures, the PC scripts executing within the Lua Processor operate in the same runtime context with access to Host Functions. Part 9a, Section 9a-5.2.1 of the standard mandates that “the host must instantiate and initialize a new Lua runtime environment” [4]. However, it provides no specifications for sandboxing or capability restrictions within that runtime. This design pattern where untrusted scripts execute with access to host-provided functions represents the attack surface examined in this paper.

The runtime trust model of S-100-based systems faces a structural turning point due to this dynamic execution architecture. The traditional trust framework of maritime information systems has focused on authenticating data sources and verifying transmission integrity for data received from trusted publishers. The latest Security Scheme (SECOM) standard aims to prevent tampering during communication through digital signature technology defined in Part 15 (Data Protection Scheme). However, integrity assurance at the transmission layer does not guarantee the functional safety of execution logic contained within that data. As noted in Section 1, the S-100 standard mandates Lua 5.1 but provides no security configurations for script execution.

Furthermore, S-100 Edition 5.2.0 Part 15 Section 15-8.2 states that “digital signatures of Feature Catalogues and Portrayal Catalogues can also be produced by some Scheme participants as required” [3]. This explicitly indicates that signature application for PCs is optional rather than mandatory. Empirical observation confirms that PC signatures remain uncommon in current practice: the IHO GI Registry distributes Product Specifications, FCs, and PCs without digital signatures; the OpenS100 reference implementation includes unsigned PC/FC files; and S-100 test datasets available through public repositories are distributed without cryptographic protection. This optional nature of PC authentication, combined with the absence of security specifications for script execution environments in Part 13, creates a critical gap where authenticated but malicious PCs could be distributed through compromised official channels without triggering security alerts. Even when signatures are employed, they only verify the authenticity of the source; they do not address the safety of the internal script logic or the execution environment. This gap between functional specification and security requirements creates a systemic vulnerability where standards-compliant implementations may inadvertently introduce risks by loading all standard Lua libraries without restriction.

### 2.2. Software Vulnerability Detection Methods

Diagnostic techniques for identifying and managing security defects in software are broadly categorized into static analysis and dynamic analysis. Static analysis examines program syntax and control flow without executing source code to identify potential weaknesses, enabling comprehensive examination of entire codebases but carrying the possibility of false positives. In contrast, dynamic analysis monitors memory corruption and exception occurrences in real-time while executing programs, accurately capturing threats during actual operation but unable to diagnose unexecuted code regions.

Security weaknesses discovered through these diagnostic processes are classified according to the Common Weakness Enumeration (CWE) standard framework [6]. CWE catalogs security weaknesses commonly occurring in software architecture, design, and code, defining specific types such as Buffer Overflow (CWE-120) and Code Injection (CWE-94). When security weaknesses develop into concrete defects exploitable by attackers, they are termed vulnerabilities, which are assessed for severity using the Common Vulnerability Scoring System (CVSS) version 3.1 [7].

CVSS provides quantitative metrics ranging from 0.0 to 10.0, evaluating vulnerabilities based on eight Base metrics including Attack Vector, Attack Complexity, Privileges Required, User Interaction, Scope, and impacts on Confidentiality, Integrity, and Availability. The CVSS Base Score combines exploitability metrics measuring ease of attack with impact metrics measuring consequences, calculated using Equation (1).

(1) $$\mathrm{BaseScore}=\mathrm{Roundup}\left(\min\left[1.08\times (ESS+ISS),10\right]\right)$$

In Equation (1), Exploitability Sub Score (ESS) is calculated from Attack Vector, Attack Complexity, Privileges Required, and User Interaction metrics, while Impact Sub Score (ISS) is derived from Scope and the three impact metrics for Confidentiality, Integrity, and Availability. Resulting scores classify vulnerabilities as None (0.0), Low (0.1 to 3.9), Medium (4.0 to 6.9), High (7.0 to 8.9), or Critical (9.0 to 10.0), providing standardized severity ratings enabling consistent assessment across different systems.

While these standardized frameworks support vulnerability classification and severity assessment, static analysis tools have inherent limitations as they rely on matching predefined patterns. Specifically, they cannot capture semantic flaws within complex logic where host languages such as C++ interact with embedded scripting languages such as Lua. For example, even when individual functions are syntactically correct, structural defects such as privilege misuse or sandboxing failures during data transfer between languages fall outside the analysis scope of tools. Therefore, expert manual code review may complement automated tools to filter thousands of basic weaknesses and identify logical flaws that contradict overall system design intent.

Furthermore, due to the distinctive nature of the maritime domain, software security in S-100 implementations directly connects to software functional safety beyond simple information protection. In navigation systems, visual representation of chart data serves as a critical information source performing essential safety functions for human navigators, including collision avoidance and grounding prevention. If malicious logic executes through portrayal engine vulnerabilities to distort depth information, omit hazard zone displays, or launch Denial of Service (DoS) attacks affecting navigation system availability, software security defects escalate into potential safety threats that could contribute to maritime incidents.

### 2.3. Related Work

Maritime cybersecurity research has grown substantially following the International Maritime Organization (IMO) MSC.428(98) resolution mandating cyber risk management in 2021 [8]. Li et al. conducted a comprehensive review of maritime cyber attacks [9]. They presented a multi-dimensional taxonomy covering ECDIS, Automatic Identification System (AIS), Global Navigation Satellite System (GNSS), Voyage Data Recorder (VDR), and Global Maritime Distress and Safety System (GMDSS), identifying third-party components and outdated operating systems as primary ECDIS vulnerabilities. However, their taxonomy focuses on network-level attacks and does not address vulnerabilities in navigation data standards requiring dynamic script execution. Longo et al. analyzed attack scenarios for manipulating maritime radar images through the ASTERIX protocol, demonstrating that navigation data standards themselves can introduce attack surfaces beyond traditional network vulnerabilities [10].

Empirical vulnerability research on operational ECDISs has documented consistent security deficiencies across manufacturers. Svilicic et al. conducted vulnerability scanning on vessels M/V Kapitan Gregorio Oca, M/V Sepen, and M/V Marko Polo [11]. They found Windows 7 systems with vulnerable Server Message Block version 1 (SMBv1) protocols still active in 2024. Jo et al. analyzed JAN-901B, Transas Navi Sailor 4000, and NACOS MULTIPILOT Platinum 2017 using the MITRE ATT&CK framework [12]. They confirmed SMB and Remote Desktop Protocol (RDP) vulnerabilities across manufacturers. These studies provide valuable documentation of protocol-level vulnerabilities but do not examine the security implications of navigation data standards themselves.

A notable gap exists in S-100 standard security research. With S-100 ECDIS voluntary adoption beginning 1 January 2026, and mandatory installation for new equipment from 1 January 2029, the literature search found limited academic work specifically addressing S-100 security vulnerabilities. While IHO has developed Part 15 (Security Scheme) and IEC 63173-2 (SECOM) establishing secure communication standards, to the best of our knowledge, independent security research validating these mechanisms or examining the security implications of Lua-based PCs remains scarce.

Recent vulnerability disclosures demonstrate that Lua sandbox implementations contain critical security flaws. The RediShell vulnerability (CVE-2025-49844), discovered at Pwn2Own Berlin 2025, represents a 13-year-old use-after-free vulnerability in Redis’s Lua scripting subsystem affecting approximately 330,000 exposed instances [13]. CVE-2022-0543 demonstrated how improper library cleanup in Redis’s Lua sandbox allowed attackers to escape via the uncleaned package global variable [14]. These CVEs illustrate the dangers of not properly managing *luaL_openlibs()* artifacts, yet no research has examined whether similar patterns exist in maritime navigation systems.

Maritime-specific vulnerability research has identified NMEA message processing as a critical attack surface. Spravil et al. developed the MANA framework for GPS spoofing detection through NMEA sentence integrity monitoring, demonstrating that application-layer protocol analysis is essential for maritime security [15]. However, their work focused on detection algorithms rather than the security of the script execution environments that process these protocols. The S-100 portrayal engine processes NMEA and other maritime protocols through Lua scripts, yet the security implications of executing untrusted Lua scripts that parse these protocols have not been examined.

Academic research on Lua security remains limited. Adámek provides comprehensive analysis of Lua sandboxing theory [16]. The thesis proposes sandbox designs based on isolating individual functions. However, the thesis does not address safety-critical system requirements or provide validation against real-world attack vectors. No published research examines Lua security in maritime symbology systems or establishes security requirements for S-100 PC implementations.

Cross-domain research in aviation and automotive safety-critical systems provides applicable frameworks. Strohmeier presents aviation cybersecurity covering Automatic Dependent Surveillance-Broadcast (ADS-B), Aircraft Communications Addressing and Reporting System (ACARS), and GPS/GNSS vulnerabilities [17]. Schmittner et al. establish FMVEA/CHASSIS methodology for joint safety-security analysis in automotive Electronic Control Units (ECUs) [18]. Ohm et al. identify dependency injection and build system compromise as primary supply chain attack vectors [19]. Additionally, Raymaker et al. identify USB-based updates as primary infiltration points in maritime systems [20]. However, none address securing external configuration files containing executable scripts in safety-critical navigation systems.

Empirical evaluations reveal significant Static Application Security Testing (SAST) tool limitations. Lipp et al. evaluated six tools against 192 known vulnerabilities [21]. They found tools miss 47–80% of vulnerabilities; combining multiple analyzers only reduces false negatives to 30–69%. Charoenwet et al. found single SAST tools warn vulnerable functions in only 52% of vulnerability-contributing commits, with FlawFinder producing 76% noise [22]. Li et al. found that only 12.7% of Java vulnerabilities were detected individually, with 70.9% undetected even combining all tools [23].

Domain-specific approaches demonstrate improved detection rates. Xiang et al. developed LuaTaint as a flow-sensitive, context-sensitive, and field-sensitive static taint analysis system targeting Lua-based web configuration interfaces in IoT firmware [24]. LuaTaint discovered 111 vulnerabilities in 2447 firmware samples with 89.29% precision, outperforming generic tools such as Semgrep. Their work demonstrates that domain-specific Lua analysis requires specialized taint propagation rules absent from general-purpose analyzers. Similarly, Costin identified that commercial tools supporting dozens of languages did not support Lua static analysis despite growing Lua codebases in mission-critical systems [25]. Systematic surveys confirm these limitations extend across programming languages; Qian et al. reviewed 78 studies on software vulnerability analysis, finding that detection performance degrades substantially when tools are applied outside their training domains [26]. No static analysis tools or research addresses maritime-specific vulnerabilities, S-100 PC security, or ECDIS configuration file analysis.

Emerging research explores Large Language Model (LLM) integration to address SAST limitations. Li et al. developed IRIS, demonstrating LLM augmentation of CodeQL and achieving 103.7% improvement in vulnerability detection by using LLMs to infer missing taint specifications that human analysts would otherwise need to write manually [27]. Lin proposed AdaTaint, combining deep learning with static taint analysis to reduce false positives by 43.7% while improving recall by 11.2% compared to traditional approaches [28]. More recently, automated query synthesis has emerged as a recommended direction: Hu et al. developed QLPro, employing a triple-voting mechanism and three-role LLM architecture to generate CodeQL vulnerability scanning rules and discovering 6 previously unknown vulnerabilities including 2 confirmed zero-days [29]. Wang et al. developed QLCoder, extending this approach by embedding LLMs in a structured synthesis loop with MCP-based reasoning constraints and achieving 53.4% success rate in synthesizing CVE-specific CodeQL queries compared to 10% for baseline approaches [30]. Bennett et al. found individual tool detection rates of only 11.2–26.5%, with combining four tools detecting only 38.8% [31]. Custom Semgrep rules improved detection to 44.7%, demonstrating domain-specific rule development remains essential. However, no research has applied LLM-assisted analysis to maritime software, representing an opportunity for future work combining neuro-symbolic approaches with S-100 domain knowledge.

### 2.4. Research Gap and Contributions

Table 1 summarizes the reviewed studies across three research categories, highlighting the systematic absence of work at the intersection of maritime navigation standards, embedded scripting security, and domain-specific vulnerability detection.

The literature review reveals three identified research gaps. First, S-100 security research appears limited despite voluntary adoption beginning January 2026 and mandatory new installations from January 2029. While Vaarandi et al. recently surveyed security monitoring methods in maritime environments, identifying key research themes and open challenges, their study focused on network-level and IoT security monitoring without examining the security implications of navigation data standards requiring script interpretation [32]. The S-100 standard mandates Lua 5.1 usage but provides no security requirements for script execution environments. Second, tools or methodologies specifically designed for detecting maritime-specific vulnerabilities in navigation data processing systems appear scarce. Recent systematic reviews confirm that maritime-specific security tools and vulnerability detection methodologies are scarce, with conventional SAST tools showing 47–80% miss rates in general software and lacking the domain knowledge necessary for detecting design-level vulnerabilities in maritime systems. Third, conventional SAST tools miss 47–80% of vulnerabilities in general software and lack the domain knowledge necessary for detecting design-level vulnerabilities in maritime systems.

This paper makes four contributions addressing these gaps. First, to the best of our knowledge, it provides one of the first security evaluations of S-100 implementations through quantitative analysis of automated tool detection rates versus expert manual review. Second, it quantifies the semantic gap in automated security analysis by contrasting automated findings with expert-identified vulnerabilities. Third, it identifies and validates through functional Proof-of-Concept (PoC) development four high-impact vulnerabilities including the Unrestricted Lua Interpreter enabling RCE on the tested platform and memory corruption flaws in S-101/S-102 data parsing. Fourth, it suggests that security verification for safety-critical maritime systems may benefit from combining automated tools with expert analysis that incorporates security expertise and maritime domain knowledge. In accordance with staged disclosure principles, this paper balances academic transparency with maritime safety obligations.

## 3. Methodology

**Figure 2:**
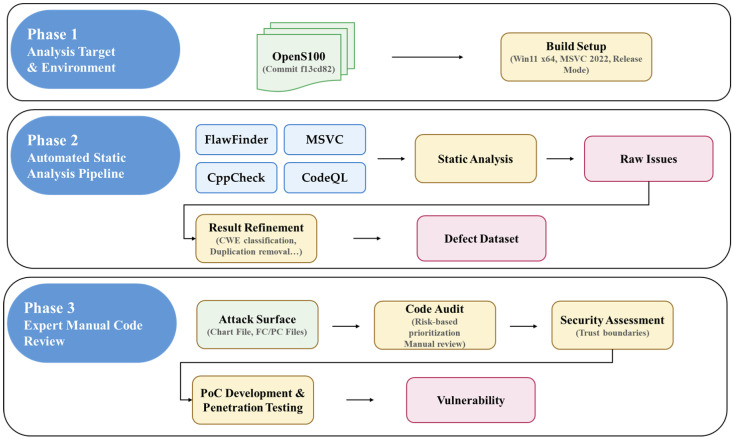
Experimental workflow showing the three-phase security analysis procedure.

This paper conducts a three-phase analysis procedure to verify the detection effectiveness of security analysis tools targeting OpenS100, an S-100 standard implementation, and to identify security blind spots arising from domain-specific logic. Figure 2 illustrates the experimental workflow comprising three sequential phases. Phase 1 establishes the analysis target and build environment. Phase 2 executes the automated static analysis pipeline where four tools generate raw issues that undergo CWE classification and deduplication to produce a refined defect dataset. Phase 3 conducts expert manual code review through attack surface identification, risk-based code audit, and security assessment of trust boundaries, culminating in PoC development and penetration testing to validate discovered vulnerabilities.

### 3.1. Analysis Target System and Environment

OpenS100, an open-source project developed based on the S-100 viewer of the Korea Hydrographic and Oceanographic Agency (KHOA) under the Ministry of Oceans and Fisheries (MOF), was selected as the analysis target for this paper [33]. OpenS100 serves as a reference implementation similar to other maritime security research testbeds such as MaCySTe, which provides a containerized environment for maritime IT/OT system experimentation [34]. However, unlike general-purpose testbeds, OpenS100 specifically implements S-100 standard compliance, with the project repository showing 18 forks indicating its use as a foundation for derivative implementations. This widespread adoption pattern means vulnerabilities identified in OpenS100 are likely to propagate to dependent systems unless developers independently implement security controls not specified in the S-100 standard.

OpenS100 is a C++-based reference implementation designed to accommodate various S-100 product specifications including S-101, the next-generation electronic navigational chart standard, as well as S-102 and S-124. The system strictly follows S-100 Part 9a Portrayal with Lua and Part 13 Scripting specifications for PC implementation. Critically, these standard specifications mandate Lua 5.1 usage but remain silent on security configurations such as library restrictions or sandboxing requirements, creating a situation where standards-compliant implementations may introduce vulnerabilities by following the specification’s functional requirements without additional security hardening. The system is released under the Mozilla Public License 2.0 [33]. This enables white-box analysis at the source code level, and the system possesses an architecture similar to commercial ECDIS, making it an appropriate model for analyzing vulnerabilities arising from standards-level design rather than implementation-specific defects. In this paper, the latest OpenS100 commit version f13cd82 was selected, which was committed on 16 December 2024.

The architecture of OpenS100 is divided into the S-100 Library for data processing and the User Interface layer for visualization, with core modules including the Feature Catalogue (FC) manager and PC processing engine. Notably, the Portrayal Engine that this paper focuses on follows the IHO LuaScriptingReference and executes Lua scripts to render charts on displays. In this process, the C++-based main program calls Lua scripts and processes execution results in a structured workflow.

The experimental environment for security analysis was established using Microsoft Visual Studio 2022 version 17.10.4 in 64-bit mode according to the official build method presented by OpenS100 developers, with the operating system being Windows 11 Education 25H2 for x64 architecture. The OpenS100 system at commit f13cd82 was built in Release mode for the x64 platform using the MSVC toolchain. Four static analysis tools were integrated into the analysis pipeline: FlawFinder version 2.0.19 for detecting security-vulnerable function calls, CppCheck version 2.18.0 for general code defect detection, Microsoft Visual C++ Code Analysis Engine with Microsoft Visual Studio 2022 version 17.10.4, and GitHub CodeQL version 2.23.8 for semantic code analysis using query-based detection. Following recommendations to strengthen the analyzer baseline with advanced semantic analysis capabilities, CodeQL was included specifically to evaluate whether query-based semantic analysis could detect the cross-language vulnerabilities that pattern-matching tools might miss. To enhance analysis precision, diagnostic procedures were performed on the pure OpenS100 implementation source code excluding external library code.

### 3.2. Automated Static Analysis Pipeline

In the first-phase diagnosis, an automated pipeline integrating multiple static analysis tools was established to identify security weaknesses throughout the source code. The tools employed are as follows. FlawFinder detects security-vulnerable function calls in C/C++ source code, evaluating functions that can cause buffer overflows or enable command injection on a risk scale of 1 to 5. CppCheck detects a broad range of programming errors including memory leaks, null pointer dereferences, and uninitialized variable usage, classifying them by severity as Error, Warning, or Style. Microsoft Visual C++ Code Analysis is a static analysis engine embedded in Visual Studio that detects buffer overruns, type mismatches, and uninitialized memory access. GitHub CodeQL employs query-based semantic analysis that constructs a relational database from source code, enabling detection of complex vulnerability patterns through declarative queries, classifying issues as Error, Warning, or Recommendation. Since each tool uses independent defect naming conventions and severity criteria, a refinement process was necessary to standardize them. In this paper, all detected defects were reclassified into categories based on security characteristics. Table 2 presents the security category definitions used in this paper.

To standardize the disparate defect naming conventions and severity criteria across tools, the following refinement process was applied. First, error codes defined independently by each tool were reclassified into security categories and assigned identification numbers according to the MITRE CWE framework. Table 3 shows representative category mapping examples for major error codes.

Second, severity criteria were unified into three levels: High, Medium, and Low. Risk levels 1 to 5 from FlawFinder, Error/Warning/Style from CppCheck, warning levels from MSVC, and Error/Warning/Recommendation from CodeQL were remapped based on defect impact and security risk. Table 4 presents the severity normalization rules for each tool.

Third, when multiple tools reported defects at the same source code location, these were consolidated into a single defect at that location, while recording the number of detecting tools as an indicator for cross-detection reliability assessment.

Finally, security-relevant defect data were secured through a filtering process that eliminated minor issues with low direct correlation to functional safety, such as simple coding style, unused code, encoding, and configuration file errors. The refinement pipeline applied systematic filtering across two stages to extract security-relevant defects from raw tool output. The four tools produced a combined total of 6052 raw issues: FlawFinder identified 125 issues, CppCheck identified 3884 issues, MSVC identified 551 issues, and CodeQL identified 1492 issues. In the first stage, trivial issues with low correlation to functional safety were excluded based on category classification. Specifically, 4261 issues were filtered out comprising CodeQuality advisories at 1778 instances, Performance warnings at 1151 instances, Unused code detections at 1023 instances, Encoding issues at 240 instances, Config warnings at 64 instances, and Deprecated function usage at 5 instances. This filtering retained 1791 security-relevant issues from the initial 6052 raw detections.

In the second stage, duplicate detections at identical source locations were consolidated. When multiple tools flagged the same file and line number, a single representative finding was selected based on maximum severity level and tool priority ordering: FlawFinder, MSVC, CodeQL, then CppCheck. This deduplication process removed 261 redundant detections, reducing the dataset from 1791 to 1530 unique defect locations. Among the deduplicated findings, 52 locations exhibited cross-tool detection where two or more tools independently identified issues at identical source positions, providing higher confidence in these findings. The final dataset comprised 49 High severity, 735 Medium severity, and 746 Low severity defect locations.

A methodological note on tool configuration: all four static analysis tools were employed with their default rulesets without custom rule development or domain-specific tuning. This configuration choice was intentional, reflecting typical security verification practices encountered in industrial software development environments where developers rely on out-of-the-box tool capabilities. The resulting “semantic gap” between automated findings and expert-identified vulnerabilities therefore represents the detection deficit developers actually encounter when using standard SAST tooling, rather than a theoretical limitation of the tools themselves. While custom CodeQL queries targeting Lua interpreter initialization patterns could potentially detect specific vulnerabilities, such customization requires prior knowledge of attack vectors, precisely the domain expertise that general-purpose tools lack and this paper demonstrates is necessary.

### 3.3. Expert Manual Code Review

In the second phase, expert manual code review was conducted to identify security blind spots in S-100 standard-specific logic that static analysis tools failed to detect. The review was performed by a PhD student with 4 years of vulnerability research experience, Offensive Security Certified Professional (OSCP) certification, and Capture The Flag (CTF) competition participation since 2010, combined with 3 years of maritime software development and 5 years of Lua programming in embedded systems.

The review scope encompassed the OpenS100 codebase at commit f13cd82, totaling 1291 source files with 101,129 lines of code (LOC) excluding external libraries. Given the complexity of exhaustive review, risk-based prioritization was employed through attack surface analysis focusing on untrusted data processing paths. The review concentrated on 842 files comprising 60,680 LOC, representing 60% of the codebase, covering the critical data processing pipeline from chart file loading through FC and PC parsing to final rendering. This scope included GISLibrary with 104 files and 21,465 LOC for ISO 8211 and HDF5 parsing, PortrayalCatalogue module with 254 files and 11,665 LOC for Lua integration, FeatureCatalog module with 135 files and 7351 LOC, S100Engine core with 93 files and 5087 LOC, and Lua portrayal rules with 256 files and 15,112 LOC. The review was conducted over 14 days with emphasis on C++ and Lua interface security within the Portrayal Engine.

The review methodology employed data flow analysis based on taint tracking principles where security vulnerabilities originate from inadequately validated external inputs. Attack surface analysis identified three primary input vectors: chart data files in S-101 *.000* and *.h5* formats, FCs and PCs consisting of XML with embedded Lua scripts, and configuration files. For each vector, taint propagation was traced from initial data reception through parsing, validation, and processing to final output, identifying weaknesses at trust boundaries.

The code audit proceeded through three core security assessments. First, sandbox analysis examined execution environment isolation to verify Lua scripts from external sources are restricted from operating system access. This verified whether dangerous Lua libraries including *os*, *io*, *debug*, and *package* are loaded during interpreter initialization, whether C++ functions exposed to Lua provide system-level capabilities, and whether sandbox mechanisms confine script privileges. Second, interface security analysis tracked all C++ to Lua bindings to verify input validation, examined type conversion vulnerabilities between languages, and identified command injection vectors through string handling. Third, privilege escalation analysis investigated whether malicious PCs could exploit trusted execution context, whether the rendering pipeline enforces data and code separation, and whether centralized PC distribution through official channels enables supply chain attacks.

Selected high-impact vulnerabilities were validated through PoC development and exploit testing. This validation generated malicious S-100 data files, specifically modified PCs embedding test scripts, and executed them in OpenS100 under controlled conditions. Four vulnerabilities demonstrating the most critical attack vectors were prioritized for PoC verification, including the Lua interpreter flaw enabling RCE and memory corruption issues in S-101/S-102 data parsing. PoCs were designed to execute benign operations such as launching calculator or notepad applications to demonstrate capability without system harm, following responsible disclosure practices. The remaining 19 vulnerabilities were identified through rigorous code audit; their detailed exploitation methods are deferred to subsequent work per the staged disclosure strategy.

## 4. Findings

Analysis results confirmed that static analysis tools demonstrate high detection rates for syntactically clear errors but show limited effectiveness in detecting defects requiring understanding of overall program operational logic and vulnerabilities based on complex data flows spanning multiple files. Automated tools identified 1530 unique defect locations, while expert review discovered 23 vulnerabilities with minimal overlap: one complete match at identical source locations, three partial matches for related defects, and 19 expert-only findings representing the majority of manual review results.

This paper employs a staged disclosure strategy aligned with coordinated vulnerability disclosure principles. Among the 23 identified vulnerabilities, four high-impact flaws were verified through functional PoC development, while the remaining 19 were identified through code audit. Detailed exploitation methods for these vulnerabilities are withheld pending remediation, balancing academic transparency with protection of safety-critical maritime infrastructure.

Table 5 presents the vulnerability summary from expert manual review, including severity classification, automated tool detection status, and PoC verification status. The Unrestricted Lua Interpreter vulnerability was undetected by all four automated tools despite enabling Remote Code Execution (RCE) on the tested platform. High-severity vulnerabilities in memory management and injection categories were similarly missed, while only simple syntactic patterns such as uninitialized members achieved partial automated detection.

Particularly, design flaws requiring understanding of S-100 standard PC mechanism, Lua script engine security configuration, maritime domain trust models, and shipping industry supply chain structures indicate that expert manual analysis is essential.

Based on these category-specific differences, the technical limitations preventing automated static analysis tools from detecting critical-level vulnerabilities are as follows. Regarding dangerous function list limitations, static analysis tools maintain lists of dangerous functions predefined by security experts. For instance, C language functions such as *strcpy()* and *gets()* are included in these lists for causing buffer overflows. However, functions that initialize and execute Lua script engines are considered normal library functions that are not inherently dangerous. The problem is that the capability to execute operating system commands within Lua scripts executed by these functions has not been removed, internal configurations that automated tools cannot verify. File-to-file data tracking limitations also constitute an important factor. The attack path for vulnerabilities comprises multiple stages. First, when navigators load chart data files they consider trustworthy into the system, the program reads script file location information from these files, then executes the script file at that location. Static analysis tools can track variable usage within a single source code file but cannot analyze such indirect connections traversing data files stored on disk to other files. Lack of understanding regarding maritime domain trust models also causes detection failures. Chart data under the S-100 standard is provided by authorized organizations such as national hydrographic offices. System developers designed systems to treat such data as trustworthy for use without separate security verification. However, if data provider organization systems are hacked or maliciously manipulated by insiders, data distributed through normal channels may contain malicious code. Such supply chain attack scenarios fall outside the realm automated tools can consider.

Finally, unverified execution environment security configuration presents an issue. Since static analysis tools analyze only source code, they cannot verify which functions are activated in script engines during program execution. For example, Lua script engines provide capabilities for operating system command execution, file system access, and network communication. Secure systems should remove these dangerous functions in advance and permit only pure computational functions, but this is determined by configuration at program execution time and cannot be verified through source code analysis alone. Despite CodeQL’s semantic analysis capabilities that enable detection of complex vulnerability patterns through query-based analysis, it also failed to detect the critical Lua interpreter vulnerability. Table 6 contrasts vulnerability types discovered by experts with types detected by all four automated tools, clearly showing detection successes and failures. Unlike Uninitialized Member where three tools succeeded and Buffer Overflow where tools partially succeeded, all four tools did not detect the Unrestricted Lua Interpreter, SQL Injection, and Path Traversal vulnerabilities in this evaluation.

## 5. Case Study: Unrestricted Lua Interpreter

This chapter provides in-depth analysis of vulnerabilities that automated static analysis tools failed to detect. Four high-impact vulnerabilities were verified through functional PoC development. First, the Unrestricted Lua Interpreter achieved RCE on the tested platform. Second, Integer Overflow in data parsing achieved DoS via heap corruption, though RCE was blocked by Windows Segment Heap. Third, Buffer Overflow achieved DoS, though RCE was blocked by /RTC compiler protection. Fourth, Integer Truncation was verified at code level and exploitation requires files exceeding 4 GB. The Lua interpreter vulnerability was selected as the primary case study for three reasons: (1) it demonstrates the highest severity (CVSS 9.3 Critical) with RCE capability on the tested platform; (2) it appears to originate from a design-level gap in the S-100 standard itself rather than implementation error; and (3) all four automated static analysis tools failed to detect it. The remaining 19 vulnerabilities were identified through rigorous code audit and follow logically similar patterns where automated tools fail to capture domain-specific security requirements. Following staged disclosure principles and security research ethics, this chapter provides only the information necessary to demonstrate the vulnerability’s existence and impact, omitting specific attack code or detailed implementation methods that could enable exploitation.

### 5.1. Vulnerability Discovery and Analysis

The S-100 standard mandates the use of Lua programming language (version 5.1) for PC implementation, as specified in Part 9a (Portrayal-Lua) and Part 13 (Scripting) of the standard [4,5]. The OpenS100 system (commit f13cd82) follows this specification by employing a Lua script engine to process PCs. PCs consist of Lua script collections that define electronic chart visualization rules, determining how each Feature in chart data is rendered on displays. Expert review confirmed that this Lua script execution environment was configured without appropriate security isolation.

The core of the vulnerability lies in the Lua interpreter initialization process within the OpenS100 portrayal engine. When initializing the Lua execution state, the system unconditionally loads all standard Lua libraries without sandboxing or capability restrictions. Listing 1 shows the vulnerable initialization sequence in the OpenS100 portrayal engine implementation.

**Listing 1.** Vulnerable Lua interpreter initialization in OpenS100.
lua_State* L = luaL_newstate();

luaL_openlibs(L); // Loads ALL standard libraries


The interpreter initialization unconditionally loads all standard Lua libraries, including *os*, *io*, *debug*, and *package* modules. This configuration exposes dangerous system-level capabilities to PC scripts without restriction. Specifically, the *os* library provides capabilities for system command execution and environment variable access; the *io* library enables arbitrary file system operations; the *debug* library grants introspection capabilities that can bypass security measures; and the *package* library allows dynamic module loading that could introduce additional attack vectors.

The IHO S-100 standard mandates Lua 5.1 usage for PC implementation but does not specify explicit security requirements for script execution environments, such as library restrictions, sandboxing requirements, or capability limitations. Part 13 (Scripting) defines the scripting mechanism and syntax requirements but remains silent on security hardening measures, leaving important security decisions to individual implementers. This standards-level gap creates a systemic vulnerability where any implementation that follows the standard’s functional requirements without adding security controls faces identical risks.

This vulnerability is classified as CWE-829 (Inclusion of Functionality from Untrusted Control Sphere) and CWE-749 (Exposed Dangerous Method or Function). The flaw resides in the system’s design decision to load external PCs without implementing proper trust boundaries or sandboxing restrictions, rather than in the failure to neutralize injected input that is subsequently evaluated as code.

### 5.2. Attack Path Analysis

This attack method can be executed through two pathways. The first pathway involves malicious PC distribution. Attackers copy legitimate PCs and insert malicious code into Lua script files. When distributed disguised as improved rendering or additional features, malicious code executes the moment users select the Catalogue and read the chart data in OpenS100 settings. Since the S-100 standard provides no mandatory digital signature or verification mechanism for PCs, users may have limited means to verify Catalogue origin and integrity. The second pathway is supply chain attack. Official S-100 PCs are provided by IHO or national hydrographic offices. If such organization systems are compromised or manipulated by insiders, large numbers of users could be affected. Given shipping and port infrastructure characteristics where identical Catalogues are widely used globally, a supply chain attack could impact multiple ECDIS installations.

**Figure 3:**
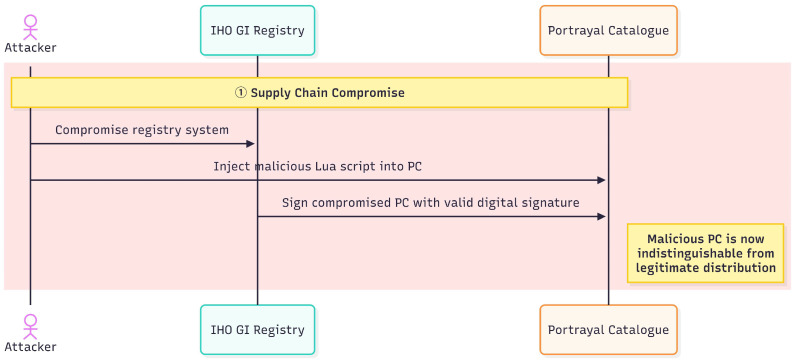
Supply chain compromise of a Portrayal Catalogue.

**Figure 4:**
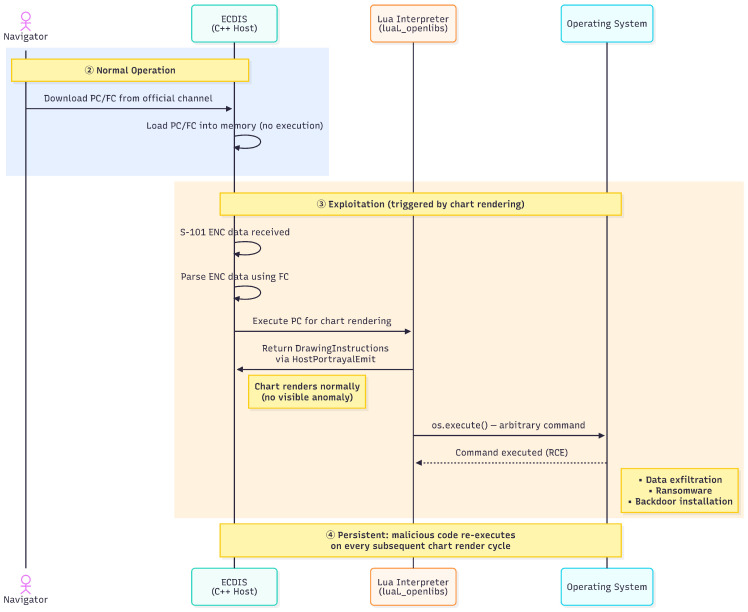
Exploitation lifecycle from PC loading through persistent command execution.

In contrast to memory vulnerability-based attacks, this vulnerability exploits legitimate interpreter functionality through the unrestricted configuration, rendering operating system security mechanisms such as Address Space Layout Randomization (ASLR), DEP, and Control Flow Guard (CFG) less effective. Lua interpreters execute as normal program components, so operating systems consider their actions legitimate. The vulnerability can achieve reliable exploitation on the tested platform without requiring memory corruption or complex exploitation techniques. Figure 3 and Figure 4 illustrate the complete attack lifecycle from supply chain compromise through persistent exploitation.

Figure 3 depicts the supply chain compromise phase involving three participants. An attacker compromises the IHO GI Registry and injects a malicious Lua script into a legitimate PC. The compromised PC then receives a valid digital signature through the standard signing process, making it indistinguishable from legitimate distributions. This phase exploits the optional nature of PC signature verification specified in S-100 Part 15 Section 15-8.2, where even signed PCs only guarantee source authenticity without validating internal script safety.

Figure 4 depicts the subsequent exploitation lifecycle involving four participants. In the normal operation phase, the navigator downloads the compromised PC from an official channel and the ECDIS loads it into memory without script execution. The exploitation phase is triggered when S-101 ENC data is received for rendering. The ECDIS parses the chart data using the FC and passes it to the Lua interpreter for portrayal processing. The interpreter simultaneously returns normal drawing instructions to the host via *HostPortrayalEmit* and executes arbitrary operating system commands through *os.execute()*.

The attack surface extends beyond process spawning to encompass multiple dangerous capabilities. Table 7 presents the complete attack surface analysis with verified exploitation vectors. Each capability was verified through PoC demonstrating actual exploitability. The *os.execute()* and *io.popen()* functions enable arbitrary command execution; *io.open()* permits file system manipulation including data exfiltration and ransomware scenarios; *os.remove()* allows destructive file operations; *debug.getinfo()* exposes runtime internals enabling sandbox escape; and *loadfile()* permits dynamic code loading from external sources.

### 5.3. Proof-of-Concept Demonstration

A minimal PoC was written and executed to verify the vulnerability’s actual impact. Following responsible disclosure principles, implementation details are omitted from this paper, with experiments conducted only to the level demonstrating vulnerability existence and impact.

**Figure 5:**
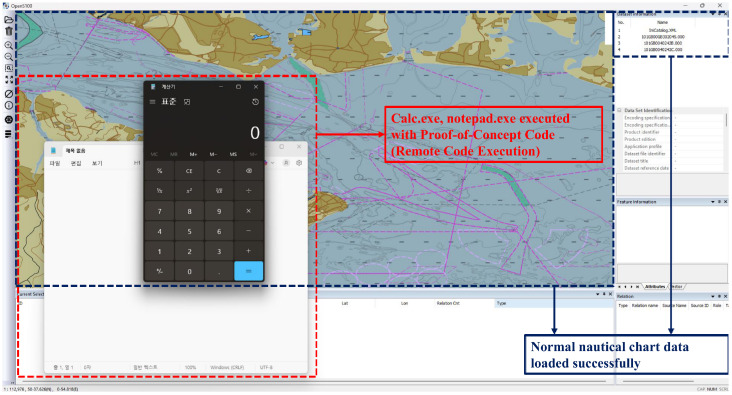
RCE demonstration via OpenS100’s unrestricted Lua interpreter.

The PoC was constructed by adding a test script to a legitimate S-101 Electronic Navigational Chart product PC. The test script was written to execute harmless programs such as calculator and notepad causing no system harm, confirming external program execution capability. The attack procedure involved two steps: first, the malicious PC was selected in OpenS100 settings; second, a legitimate S-101 chart file was opened. Experimental results showed that the specified programs executed immediately upon opening the chart file on Windows 11, demonstrating RCE capability where malicious PCs can be distributed through official channels or supply chain compromise to achieve code execution on target systems. Figure 5 illustrates the attack execution on the tested Windows 11 configuration.

Upon loading the S-101 chart file after selecting the compromised PC, the embedded Lua script executes operating system commands, launching the calculator application as specified by the attacker. Critically, the chart data is rendered normally on the display, showing the expected maritime features and depth information, making the malicious activity undetectable to users during routine operations.

Key characteristics confirmed through experimentation on the tested Windows 11 configuration are as follows. First, the vulnerability exhibits reliable exploitation on the tested platform. Second, platform independence exists at the design level since the vulnerability resides in the Lua interpreter initialization configuration rather than platform-specific code. Third, detection difficulty exists since malicious scripts exist as normal Lua files, making recognition as malicious code challenging for antivirus software or intrusion detection systems. Fourth, operational stealth exists since the ECDIS continues to function normally from the user’s perspective, rendering charts correctly while malicious operations proceed in the background.

### 5.4. Impact Assessment

This vulnerability may have substantial impacts given the characteristics of ECDISs used in the shipping industry. ECDIS are core systems for vessel navigation safety, mandatorily installed on all merchant vessels above a certain size according to IMO Safety of Life at Sea (SOLAS) convention [35]. If attackers gain control of ECDISs, navigation safety could be affected. Chart information could potentially be manipulated to display incorrect data, or hazard information could be hidden, potentially leading to navigation incidents. Since chart rendering proceeds normally during attack execution as demonstrated in the PoC, navigators may have limited indication that displayed information has been compromised, potentially continuing to make navigation decisions based on altered data. This stealth characteristic distinguishes the vulnerability from traditional cyber attacks that typically generate observable system anomalies.

Implications regarding information leakage also warrant consideration. Sensitive operational information such as vessel position data, route data, and cargo information could potentially be exfiltrated. Such information could be exploited for various purposes including competitive intelligence or tracking specific cargo movements. For military-purpose vessels or vessels carrying strategic materials, security considerations may apply. The persistent nature of the compromise, combined with normal system operation, could enable long-term intelligence gathering operations that may be difficult to detect through conventional security monitoring.

Impacts on port infrastructure also warrant consideration. Compromising port facility ECDISs could affect vessel entry and departure management operations. Modern ports handle simultaneous entry and departure of multiple vessels, managing safe berthing and unberthing through ECDIS. System compromise or data manipulation could affect port operations, potentially impacting logistics systems. The demonstrated capability to deploy ransomware could target port operations, causing operational disruption.

Supply chain attack through centralized PC distribution represents an additional consideration. Since S-100 PCs are distributed centrally by IHO and national hydrographic offices, a supply chain compromise could potentially impact multiple vessels and port facilities. The combination of centralized distribution, optional digital signatures for PCs as specified in S-100 Part 15, and the stealth characteristics demonstrated in the PoC could create conditions for supply chain attacks that may operate undetected, warranting attention from maritime security stakeholders.

The vulnerability was assessed using CVSS version 3.1 with the Base Score formula in Equation (1) to provide standardized severity quantification.

The Attack Vector is set to Network (AV:N) because PCs are distributed through online channels including the IHO GI Registry, Regional ENC Coordinating Centers (RENCs), and national hydrographic office web services. While ECDISs may appear to operate on isolated networks, the S-100 ecosystem increasingly relies on network-based distribution infrastructure. The emerging S-100 Security Exchange Scheme (SECOM) defined in Part 15 explicitly addresses secure network transmission of chart data, confirming the industry’s trajectory toward online distribution. Physical media (AV:P) or local access (AV:L) would only apply if updates were exclusively delivered via USB drives without any network connectivity, a scenario inconsistent with modern maritime digitalization initiatives. Under current deployment practices requiring manual user download and installation, the vulnerability receives a base score of 9.3, Critical with vector string CVSS:3.1/AV:N/AC:L/PR:N/UI:R/S:C/C:H/I:H/A:H. Unlike memory corruption vulnerabilities that modern operating systems may partially mitigate through ASLR and DEP, this design-level flaw exploits legitimate Lua functionality, rendering conventional exploit mitigation techniques ineffective. If the maritime industry transitions to automatic PC update mechanisms where ECDISs automatically download and apply updates without user intervention, the User Interaction metric changes from Required to None (UI:N), escalating the score to 10.0, Critical, representing the maximum possible CVSS severity. This potential escalation underscores the critical importance of implementing mandatory security controls in S-100 specifications before automatic update infrastructure becomes operational.

The Scope metric is set to changed (S:C) because successful exploitation impacts resources beyond the vulnerable component’s security authority. The ECDIS application operates within its designated security context, but successful RCE through the Lua interpreter allows attackers to execute arbitrary commands at the operating system level, affecting the entire host system rather than just the ECDIS process. This cross-boundary impact is evidenced by PoC demonstrations where calculator and notepad applications, which are external to the ECDIS security domain, were launched through the vulnerability. The changed scope designation follows CVSS 3.1 specification guidance that scope changes when a vulnerability in one component enables impact on components outside its security authority.

### 5.5. Patch Validation and Mitigation

To verify the feasibility of mitigating the identified vulnerability, a secure Lua interpreter configuration was implemented and validated. The implemented patch employs the post-initialization removal approach to maintain compatibility with existing PC implementations while addressing the security vulnerability. Listing 2 shows the secure configuration that disables dangerous Lua libraries after initialization.

**Listing 2.** Secure Lua interpreter configuration disabling dangerous libraries.
m_l = luaL_newstate ();

luaL_openlibs (m_l);

// [SECURITY PATCH] Disable dangerous libraries

luaL_dostring (m_l,

    "os = nil "

    "io = nil "

    "debug = nil "

    "package.loadlib = nil "

    "package.cpath = ’’ "

    "loadfile = nil "

    "dofile = nil "

    "load = nil "

    "rawset = nil "

    "loadstring = nil"

);


The patch blocks operating system command execution, file system access, debugging capabilities, native library loading, and dynamic code evaluation. Table 8 summarizes the blocked capabilities and their associated risks.

#### 5.5.1. Execution Order and Rawget Bypass Prevention

The library removal timing is critical for security. A potential reviewer concern involves *rawget()* bypass attempts where malicious scripts might attempt to access removed libraries through low-level table operations. The patch design prevents this attack vector through strict execution ordering.

The Lua interpreter initialization follows a deterministic sequence: (1) *luaL_newstate()* creates the Lua state, (2) *luaL_openlibs()* loads all standard libraries including *os*, *io*, and *debug*, (3) *luaL_dostring()* executes the security patch setting library references to *nil*, and (4) external PC scripts are loaded via *load_file()*. Because the patch executes *before* any external script loads, library references are already removed from the global table (*_G*) when malicious scripts attempt access.

Consequently, bypass attempts such as *rawget(_G, "os")* return *nil* because the reference was eliminated in step (3) before the script executed in step (4). This ordering guarantee makes the patch resistant to *rawget*/*rawset* bypass techniques, *setfenv*/*getfenv* environment manipulation, and metatable-based *__index* interception.

#### 5.5.2. Validation Testing Results

Empirical validation confirmed complete RCE mitigation while preserving PC compatibility. Table 9 summarizes the test results.

The patch preserves essential Lua capabilities required by S-101 PCs including *require* for module loading, *rawget* for metatable-safe table access within *__index* metamethods, and standard libraries (*math*, *string*, *table*, *pairs*/*ipairs*) for portrayal rule computation. The *rawset* function is proactively blocked as a defense-in-depth measure: static analysis of the S-101 reference PC confirmed zero *rawset* invocations across all 256 Lua source files, while its capability to bypass *__newindex* metamethods represents a potential sandbox evasion vector. This selective blocking approach enables security hardening without breaking legitimate PC functionality.

The proposed mitigation introduces negligible performance overhead. The library removal patch executes a single *luaL_dostring()* call during interpreter initialization, performing O(1) operations to set dangerous library references to *nil*. This occurs once at startup before any portrayal script execution; the patch does not affect runtime portrayal performance. No measurable impact on startup time or memory consumption was observed during validation testing, though formal benchmarking in resource-constrained embedded ECDIS environments remains a recommended direction for production deployment validation.

#### 5.5.3. Security and Standard Functional Compatibility

A potential concern is whether disabling Lua standard libraries conflicts with the extensibility intended by the S-100 standard. Analysis of Part 9a demonstrates that this conflict does not arise under the current architecture. The S-100 Part 9a portrayal model defines a closed-loop execution environment where all data exchange between Lua scripts and the host system occurs exclusively through host callback functions [4]. Feature data is not directly exposed to portrayal scripts; instead, scripts request attributes, spatial relations, and information associations via host callbacks, and return drawing instructions to the host via *HostPortrayalEmit* [4]. Section 9a-7 explicitly states that the portrayal does not use a data input schema, with all data passed through the Part 13 host interface. This architecture means that Lua scripts have no legitimate requirement for direct file system access (*io*), operating system command execution (*os*), runtime introspection (*debug*), or dynamic native library loading (*package.loadlib*). The disabled libraries fall entirely outside the functional scope defined by Part 9a.

Empirical validation confirms this architectural analysis. The proposed patch was tested against the S-101 reference PC distributed with OpenS100, which comprises 256 files and 15,112 lines of Lua code. All portrayal functions executed successfully with the dangerous libraries disabled, producing identical rendering output. The compatibility boundary was identified through systematic testing of low-level table operations. *rawset()* is not used in the S-101 reference PC and was disabled as a defense-in-depth measure, as it enables *__newindex* metamethod bypass that could permit unauthorized modification of host-registered callback functions. *rawget()* required preservation: static analysis of the S-101 reference PC identified 13 *rawget()* invocations across 6 source files, of which 2 invocations (15%) are structurally mandatory within *__index* metamethods to prevent infinite recursion in the inheritance chain implementation, while 11 invocations (85%) serve as convenience usage to bypass lazy-loading side effects. Disabling *rawget()* caused PC loading failures, confirming that this function must be preserved for current PC compatibility, as discussed in Section 6.2.

Should future S-100 revisions require capabilities beyond pure computation, such as metadata reading from external sources or modular script loading, the appropriate mechanism is defining new host callback functions rather than exposing Lua standard libraries. This approach is consistent with the Part 9a design philosophy where the host mediates all external interactions, enabling fine-grained access control. For example, a controlled *HostReadMetadata* callback could provide specific metadata access without granting unrestricted file system operations. This callback-based extensibility model preserves the security boundary while accommodating evolving functional requirements.

## 6. Limitations and Discussion

This section addresses methodological constraints and outlines future research directions for maritime software security.

### 6.1. Limitations

This paper analyzed a single reference implementation, OpenS100 at commit f13cd82, rather than multiple commercial ECDISs. This constraint arises from: (1) proprietary nature of commercial ECDIS software limiting source code access, (2) resource constraints preventing procurement and analysis of multiple licensed systems, and (3) regulatory restrictions on reverse engineering safety-critical maritime equipment. While direct experimentation on commercial ECDIS platforms was not conducted due to security licensing and intellectual property restrictions, the generalization argument rests on OpenS100’s official status within the IHO S-100 infrastructure. OpenS100 is listed as an official interim S-100 infrastructure component in IHO document HSSC16-04.5A, Annex 2, and was recognized by the S-100 Working Group as “a key component in validating the practical implementation of S-100 standards” at S-100WG10 in September 2025 [36]. The project is maintained by KHOA under IHO oversight and uses the official IHO LuaScriptingReference for portrayal catalogue implementation. Its 18 repository forks on GitHub indicate widespread use as a foundation for derivative systems. Since the identified vulnerabilities originate from architectural gaps in S-100 standard specifications Parts 9a and 13 rather than OpenS100-specific implementation errors, any standards-compliant implementation that faithfully follows the same specifications faces identical architectural risks unless developers independently implement security controls not mandated by the standard. However, commercial ECDIS vendors may have implemented proprietary security hardening measures beyond standard requirements, such as Lua library restrictions or process-level sandboxing, that mitigate the identified risks in their specific deployments. Verification of such vendor-specific countermeasures across the commercial ECDIS ecosystem remains an open research question requiring industry collaboration.

The findings generalize beyond the specific S-101 product specification to the broader S-100 family. OpenS100 implements a shared Lua interpreter initialization architecture used across multiple S-100 product specifications including S-101 (Electronic Navigational Charts), S-102 (Bathymetric Surface), and S-124 (Navigational Warnings). All these specifications inherit the same portrayal engine architecture defined in S-100 Part 9a and Part 13, meaning they share identical *luaL_openlibs()* initialization patterns. Consequently, the Unrestricted Lua Interpreter vulnerability and the proposed mitigation patch apply uniformly across the S-100 product family rather than being S-101-specific.

Exploitation verification was conducted for 4 of 23 identified vulnerabilities (17% coverage), achieving RCE on the tested platform via the Lua interpreter flaw and DoS via memory corruption in data parsing modules. The remaining 19 vulnerabilities, including SQL Injection and Path Traversal patterns, were identified through rigorous code audit where clear vulnerability sinks were confirmed in source code analysis. This selective PoC strategy reflects three considerations: (1) ethical constraints requiring avoidance of unintended damage to operational systems or databases during SQL injection testing, (2) diminishing returns where vulnerabilities with well-established exploitation patterns do not require individual PoC demonstration when code review confirms the presence of unsanitized user input reaching dangerous sinks, and (3) research prioritization concentrating resources on the Critical-severity Unrestricted Lua Interpreter vulnerability representing this paper’s primary contribution.

The expert manual review was conducted by a single researcher, introducing potential single-auditor bias. While cross-validation by multiple independent experts would strengthen confidence in the completeness of the findings, this limitation is mitigated by several structural factors. First, the review employed a systematic methodology rather than ad hoc inspection: attack surface identification defined three explicit input vectors, namely chart data files, FC/PC scripts, and configuration files, taint propagation was traced from data reception through parsing to output for each vector, and three structured security assessments (sandbox analysis, interface security analysis, and privilege escalation analysis) were applied as described in Section 3. Second, all identified vulnerabilities were classified using the MITRE CWE framework and assessed with standardized CVSS metrics, reducing subjectivity in severity evaluation. Third, the four highest-impact vulnerabilities were validated through functional PoC development producing independently reproducible results, including CVE-assigned RCE demonstration, which constitutes objective evidence rather than subjective judgment. Fourth, the main contributor of the OpenS100 project independently acknowledged the identified vulnerabilities during the coordinated disclosure process. The remaining 19 vulnerabilities identified solely through code audit carry inherent risk of false positives; their validation through PoC development is deferred to subsequent work per the staged disclosure strategy. Future studies would benefit from multi-expert cross-validation to further reduce potential omission bias.

Four representative static analysis tools, namely FlawFinder, CppCheck, MSVC, and CodeQL, were employed with their default rulesets, intentionally reflecting typical security verification practices in industrial software development environments. This methodological choice warrants explicit justification: the study objective is to measure how effectively domain-agnostic automated tools detect maritime-specific vulnerabilities when used by typical system developers rather than security specialists. The resulting “semantic gap” between automated findings and expert-identified vulnerabilities represents the detection deficit that developers actually encounter when relying on out-of-the-box tool capabilities without custom rule development.

### 6.2. PC Security Configuration Gap

Implementation of the Unrestricted Lua Interpreter mitigation patch revealed an unanticipated standards-level security gap that extends beyond the study’s original scope. This finding demonstrates how security implementation efforts can uncover previously undocumented specification issues requiring standards-body intervention.

Static analysis of the S-101 reference PC distributed with OpenS100 identified 13 *rawget()* invocations across 6 source files with two distinct usage patterns. Analysis reveals 11 of 13 uses (85%) serve convenience rather than technical necessity, while 2 uses (15%) are structurally mandatory within the metatable *__index* implementation.

The S-101 reference PC implements object inheritance through Lua’s metatable mechanism. Within *__index* metamethods, standard table access creates infinite recursion because accessing a nonexistent key triggers the same *__index* function. The *rawget()* function bypasses this metamethod invocation, enabling direct table storage access without recursion. This represents a structural requirement of Lua’s metamethod system rather than an implementation choice.

S-100 Edition 5.2.0 represents the authoritative specification for maritime geospatial data standards. Analysis of Parts 9a (Portrayal) and 13 (Scripting) reveals significant specification gaps. The standard specifies Lua version 5.1 usage but does not specify which Lua standard libraries are permitted or prohibited, security hardening requirements for script execution, API restrictions or capability controls, or sandboxing and isolation requirements.

Based on identified gaps, the following addition to S-100 Part 9a is proposed as Section 9a-7 (PC Security Requirements). First, prohibited low-level table operations: PC scripts shall not use functions bypassing Lua’s standard table access semantics including *rawget()*, *rawset()*, *rawequal()*, and *rawlen()*. Second, prohibited metatable operations: PC scripts shall not define or modify metatables on objects not owned by the script. Third, mandated object model: Feature inheritance shall be implemented through explicit delegation methods or composition patterns; metatable-based automatic delegation is prohibited. Fourth, conformance verification: IHO PC distribution shall include static analysis verification that PCs contain no invocations of prohibited functions and implement object models using permitted patterns.

### 6.3. Pathway to Standardization

The findings of this study have practical pathways for reaching international organizations responsible for electronic chart and ECDIS standards. The identified vulnerability has been assigned a Common Vulnerabilities and Exposures (CVE) identifier through the MITRE/CVE program, establishing a permanent public record accessible to ECDIS manufacturers, classification societies, and maritime regulatory bodies worldwide. CVE registration ensures that the vulnerability is indexed in the National Vulnerability Database (NVD) and incorporated into commercial vulnerability management workflows used by equipment vendors.

In accordance with coordinated vulnerability disclosure principles, the authors met with the main contributor of the OpenS100 project to present the research findings and propose the mitigation patch described in Section 5. The contributor acknowledged the identified vulnerability and agreed to integrate the security patch into the official repository. The patch is scheduled for upstream commit following acceptance of this paper and prior to publication, ensuring that the remediation is available to all downstream implementations before public disclosure of detailed exploitation methods. This coordinated approach balances academic transparency with responsible protection of operational maritime systems.

Beyond public disclosure and developer coordination, the authors are members of an IHO S-129 Project Team (Under Keel Clearance Management), an S-100 based product specification, providing direct institutional access to IHO’s S-100 standards development process. The authors also regularly participate in S-100 Working Group (S-100WG) meetings both in person and online. This involvement enables the research findings to be communicated to the S-100WG through established IHO reporting mechanisms. Furthermore, the analyzed OpenS100 system is maintained by KHOA under MOF, which serves as the Republic of Korea’s national hydrographic office and an active IHO Member State. This institutional relationship provides an additional pathway for conveying security recommendations to the S-100 standards development community.

Several factors support the likelihood that the findings will be considered by relevant stakeholders. First, the S-100 ECDIS mandatory adoption deadline of January 2029 provides a window for incorporating security controls into standard revisions before widespread deployment. Second, the identified vulnerability originates from specification gaps in the S-100 standard itself rather than implementation-specific errors, meaning that remediation fundamentally requires standards-level intervention by IHO. Third, the International Association of Classification Societies (IACS) Unified Requirements E26 and E27 [37,38], which mandate cyber resilience for ships and onboard systems, create regulatory expectations that classification societies may reference when evaluating S-100 ECDIS type approval. The convergence of an approaching regulatory deadline, a standards-level root cause, and an existing cybersecurity regulatory framework establishes conditions favorable for the findings to inform future S-100 revisions.

### 6.4. Future Research

The following research directions are recommended.

*(1) Multi-Vendor ECDIS Verification:* Comprehensive ecosystem assessment requires analyzing commercial ECDIS implementations from multiple vendors to determine whether identified vulnerabilities represent systemic patterns or isolated occurrences. Such research requires industry collaboration and regulatory support to overcome proprietary code access constraints, similar to IACS Unified Requirements E26 and E27 for maritime cybersecurity introduced in 2022 [37,38].

*(2) PC Vetting Pipeline:* Development of automated security verification infrastructure for PC distribution through IHO channels, comprising static analysis detecting prohibited API usage, dynamic sandboxed execution for behavioral analysis, cryptographic signing for authenticated distribution, and continuous monitoring for post-distribution vulnerability disclosure.

*(3) Maritime-Specific Security Tools:* Development of domain-aware static analysis tools incorporating S-100 trust models and maritime functional safety requirements. While general-purpose tools like CodeQL provide strong technical capabilities, they lack maritime domain knowledge necessary for detecting design-level vulnerabilities like the Unrestricted Lua Interpreter flaw.

*(4) S-100 Security Certification Framework:* Development of security evaluation criteria for S-100 implementations, analogous to Common Criteria for information security or DO-178C for avionics software, providing standardized assurance levels for ECDIS deployments.

*(5) Large Language Model Augmented Vulnerability Detection:* Recent advances in LLM-augmented static analysis present promising directions for addressing the semantic gap between automated tools and expert manual review identified in this study. Large Language Models trained on extensive code corpora demonstrate capability for understanding cross-language semantics, inferring implicit security constraints from specification documents, and generating domain-specific analysis rules from natural language descriptions, suggesting potential for overcoming the limitations observed in traditional static analysis approaches.

## 7. Conclusions

This paper evaluated the vulnerability detection capabilities of automated static analysis tools versus expert manual review targeting the OpenS100 implementation. While automated tools identified numerous syntactic defects, they failed to detect the majority of expert-identified vulnerabilities, including the PoC-verified Unrestricted Lua Interpreter flaw enabling RCE on the tested platform. These results indicate that current functional safety certification frameworks require supplementation to address design-level cyber risks arising from structural gaps in the S-100 standard specification.

Accordingly, the experimental evidence supports that IHO mandate the following security controls in future standard revisions: (1) process-level sandboxing through operating system mechanisms, (2) mandatory digital signatures and verification for PCs and FCs beyond chart data alone, and (3) defense-in-depth architecture ensuring multiple independent security layers. These recommendations align with established security principles from safety-critical domains including aviation and automotive.

As maritime digital transformation accelerates, security must be integrated from the design phase. This paper emphasizes that combining automated efficiency with domain-specific expertise is essential for building secure maritime infrastructure protecting global navigation safety. In accordance with the staged disclosure strategy, future work will present detailed patch logic for the remaining vulnerabilities and real-time security verification algorithms to support practical security hardening of the S-100 ecosystem.

## Figures and Tables

**Table 1:** Summary of Reviewed Studies and Identified Gaps.

Research Focus	Representative Studies	Key Limitation
ECDIS Security	[9,11,12]	Network-level only; no data standard security
S-100 Standard Security	Limited literature	Insufficient coverage despite 2029 mandate
Lua Sandbox Security	[13,16]	No maritime symbology application
Safety-Critical Systems	[17,18]	No embedded scripting frameworks
SAST Limitations	[21,22,23]	General vulnerabilities; 47–80% miss rates
Domain-Specific Analysis	[24,28]	IoT/general domains; no maritime tools
LLM-Assisted SAST	[27,29,30]	General code; no maritime application

**Table 2:** Security Category Definitions.

Category	Definition	Example Issues
Memory	Memory management errors	Buffer Overflow, Memory Leak, Use-After-Free
Injection	Malicious command injection	SQL Injection, Script Injection, Command Injection
NullPointer	NULL pointer dereference	Uninitialized pointer usage
TypeSafety	Data type mismatch errors	Integer Overflow, Improper Type Casting
Initialization	Variable initialization omission	Uninitialized member variable usage
Input Validation	Lack of input validation	Path validation omission, File size validation absence
Logic	Program logic errors	Incorrect conditional statements, Resource leak
Concurrency	Concurrency handling errors	Race condition, Deadlock
Other	Other security issues	Configuration errors, Other weaknesses

**Table 3:** Representative Category Mapping Examples.

Tool	Origin Code	Mapped Category	CWE
FlawFinder	CWE-78	Injection	CWE-78
FlawFinder	CWE-119	Memory	CWE-119
FlawFinder	CWE-362	Concurrency	CWE-362
CppCheck	nullPointer	NullPointer	CWE-476
CppCheck	memleak	Memory	CWE-401
CppCheck	uninitMemberVar	Initialization	CWE-457
MSVC	C6386	Memory	CWE-787
MSVC	C6387	NullPointer	CWE-476
MSVC	C26495	Initialization	CWE-457
CodeQL	Inconsistent null	NullPointer	CWE-476
CodeQL	Stack memory	Memory	CWE-562
CodeQL	Constant return	Other	CWE-398

**Table 4:** Severity Normalization Rules.

Tool	Original Severity	Normalized	Rationale
FlawFinder	Risk Level 4-5	High	Exploitable vulnerabilities
FlawFinder	Risk Level 3	Medium	Potential security issues
FlawFinder	Risk Level 1-2	Low	Minor concerns
CppCheck	Error	High	Critical errors
CppCheck	Warning	Medium	Potential bugs
CppCheck	Style, Performance	Low	Code quality issues
MSVC	C6386, C6387	High	Memory/NULL pointer
MSVC	C26495, C4244	Medium	Init./conversion
MSVC	C4819, C4101	Low	Encoding/unused
CodeQL	Error	High	Semantic defects
CodeQL	Warning	Medium	Potential issues
CodeQL	Recommendation	Low	Code quality

**Table 5:** Summary of Identified Vulnerabilities and PoC Status.

No.	Vulnerability	Severity	Detection	PoC	Disclosure
1	Lua Interpreter (CWE-829)	9.3 (Crit)	Expert	Yes (RCE)	Full (Section 5)
2	Integer Overflow (CWE-190)	7.8 (High)	Expert	Yes (DoS) ^b^	Restricted ^a^
3	Buffer Overflow (CWE-120)	7.8 (High)	Tools/Expert	Yes (DoS) ^c^	Restricted ^a^
4	Integer Truncation (CWE-681)	4.3 (Med)	Expert	Yes (Code) ^d^	Restricted ^a^
5–23	Remaining 19 vulnerabilities (2.6–7.6)			Restricted ^a^	Restricted ^a^

^a^ PoC deferred per CVD; details classified as “Controlled Distribution.” ^b^ RCE blocked by Windows Segment Heap. ^c^ RCE blocked by /RTC compiler protection. ^d^ Code-level verification; exploitation requires 4 GB+ file.

**Table 6:** Detection Capability by Vulnerability Type.

Vulnerability Type	Det.	Reason for Failure
Unrestricted Lua Interpreter (CWE-829, 749)	0/4	Script engine APIs not flagged
SQL Injection (CWE-89)	0/4	Dynamic query not traced
Path Traversal (CWE-22)	0/4	Path validation not analyzed
Use-After-Free (CWE-416)	1/4	Complex lifetime analysis
Integer Overflow (CWE-190)	0/4	Cross-function overflow not traced
Uninitialized Member (CWE-457)	3/4	Simple syntactic pattern
Buffer Overflow (CWE-120)	2/4	Detected by MSVC, CodeQL

**Table 7:** Attack Surface Analysis of Unrestricted Lua Libraries.

Function	Capability	Attack Scenario
os.execute()	Command execution	RCE, malware deployment
io.popen()	Process spawning	Backdoor, reverse shell
io.open()	File system access	Data exfiltration, ransomware
os.remove()	File deletion	Data destruction, DoS
debug.getinfo()	Runtime introspection	Sandbox escape, privilege escalation
loadfile()	Dynamic code loading	Payload staging, persistence
os.getenv()	Environment access	Credential harvesting
package.loadlib()	Native library loading	Arbitrary code execution

**Table 8:** Blocked Capabilities and Risk Mitigation.

Target	Risk Factor	Status
os	execute, exit, remove, rename	Blocked
io	open, popen, read, write	Blocked
debug	Sandbox bypass capabilities	Blocked
package.loadlib	Native DLL loading	Blocked
package.cpath	C library search path	Empty string
loadfile/dofile	External file execution	Blocked
rawset	__newindex bypass, table tampering	Blocked
load/loadstring	Dynamic code evaluation	Blocked

**Table 9:** Patch Validation Test Results.

Test Scenario	Expected Behavior	Result
Direct os.execute()	Returns nil, no execution	Pass
rawget(_G, “os”) bypass	Returns nil, bypass fails	Pass
Malicious PC loading	RCE blocked, chart renders	Pass
Normal PC loading	Chart rendering succeeds	Pass
Feature processing	Visualization rules execute	Pass
System stability	No crashes or errors	Pass

## Data Availability

Data available on request due to restrictions (e.g., privacy, legal or ethical reasons). The OpenS100 source code is publicly available at https://github.com/S-100ExpertTeam/OpenS100 (accessed on 16 December 2025).

## Abbreviations

The following abbreviations are used in this manuscript:

ACARS	Aircraft Communications Addressing and Reporting System
ADS-B	Automatic Dependent Surveillance-Broadcast
AIS	Automatic Identification System
ASLR	Address Space Layout Randomization
CFG	Control Flow Guard
CTF	Capture The Flag
CVE	Common Vulnerabilities and Exposures
CVSS	Common Vulnerability Scoring System
CWE	Common Weakness Enumeration
DEF	Drawing Exchange Format
DEP	Data Execution Prevention
DoS	Denial of Service
ECDIS	Electronic Chart Display and Information Systems
ECU	Electronic Control Unit
ENC	Electronic Navigational Chart
FC	Feature Catalogue
GMDSS	Global Maritime Distress and Safety System
GNSS	Global Navigation Satellite System
HDF5	Hierarchical Data Format version 5
IACS	International Association of Classification Societies
IHO	International Hydrographic Organization
IMO	International Maritime Organization
IoT	Internet of Things
KHOA	Korea Hydrographic and Oceanographic Agency
LLM	Large Language Model
LOC	Lines of Code
MOF	Ministry of Oceans and Fisheries
MSVC	Microsoft Visual C++
NMEA	National Marine Electronics Association
OSCP	Offensive Security Certified Professional
PC	Portrayal Catalogue
PoC	Proof of Concept
RCE	Remote Code Execution
RDP	Remote Desktop Protocol
RENC	Regional ENC Coordinating Center
SAST	Static Application Security Testing
SECOM	Secure Communication Between Ship and Shore
SMBv1	Server Message Block version 1
SOLAS	Safety of Life at Sea
VDR	Voyage Data Recorder
